# Bioinformatic prediction of immunodominant regions in spike protein for early diagnosis of the severe acute respiratory syndrome coronavirus 2 (SARS-CoV-2)

**DOI:** 10.7717/peerj.11232

**Published:** 2021-04-08

**Authors:** Siqi Zhuang, Lingli Tang, Yufeng Dai, Xiaojing Feng, Yiyuan Fang, Haoneng Tang, Ping Jiang, Xiang Wu, Hezhi Fang, Hongzhi Chen

**Affiliations:** 1 Department of Laboratory Medicine, The Second Xiangya Hospital, Central South University, Changsha, Hunan, China; 2 Department of Parasitology, Xiangya School of Basic Medicine, Central South University, Changsha, Hunan, China; 3 Key Laboratory of Laboratory Medicine, Ministry of Education, Zhejiang Provincial Key Laboratory of Medical Genetics, College of Laboratory Medicine and Life Sciences, Wenzhou Medical University, Wenzhou, Zhejiang, China; 4 National Clinical Research Center for Metabolic Disease, Key Laboratory of Diabetes Immunology, Ministry of Education, Metabolic Syndrome Research Center, and Department of Metabolism & Endocrinology, The Second Xiangya Hospital, Central South University, Changsha, Hunan, China

**Keywords:** SARS-CoV-2, Spike protein, Antigen-capture, Immunodominant fragments, COVID-19

## Abstract

**Background:**

To contain the pandemics caused by SARS-CoV-2, early detection approaches with high accuracy and accessibility are critical. Generating an antigen-capture based detection system would be an ideal strategy complementing the current methods based on nucleic acids and antibody detection. The spike protein is found on the outside of virus particles and appropriate for antigen detection.

**Methods:**

In this study, we utilized bioinformatics approaches to explore the immunodominant fragments on spike protein of SARS-CoV-2.

**Results:**

The S1 subunit of spike protein was identified with higher sequence specificity. Three immunodominant fragments, Spike_56-94_, Spike_199-264_, and Spike_577-612_, located at the S1 subunit were finally selected via bioinformatics analysis. The glycosylation sites and high-frequency mutation sites on spike protein were circumvented in the antigen design. All the identified fragments present qualified antigenicity, hydrophilicity, and surface accessibility. A recombinant antigen with a length of 194 amino acids (aa) consisting of the selected immunodominant fragments as well as a universal Th epitope was finally constructed.

**Conclusion:**

The recombinant peptide encoded by the construct contains multiple immunodominant epitopes, which is expected to stimulate a strong immune response in mice and generate qualified antibodies for SARS-CoV-2 detection.

## Introduction

The severe acute respiratory syndrome coronavirus 2 (SARS-CoV-2) is highly contagious and has caused more than one hundred million infection cases and over 2.4 million deaths (https://www.who.int/, as of February 15, 2021), posing a huge economic and social burden internationally (Lan et al., 2020; Shang et al., 2020). The reports of SARS-CoV-2 reinfection cases suggest that stronger international efforts are required to prevent COVID-19 re-emergence in the future (Zhan, Deverman & Chan, 2020). Nevertheless, the possibility of SARS-CoV-2 becoming a seasonal epidemic cannot be excluded (Shaman & Galanti, 2020). Even worse, the large number of asymptomatic infections greatly increase the difficulties of epidemic control (Rothe et al., 2020). At present, no specific drugs have been developed for SARS-CoV-2, and the effectiveness of the vaccines on the market still needs time to be evaluated. Therefore, early detection and isolation of infected people are still indispensable means to control the spread of the epidemic, which requires accurate, early, economical, and easy-to-operate diagnostic methods (Yan, Chang & Wang, 2020).

The real-time reverse transcriptase-polymerase chain reaction (RT-PCR) and antibody-capture serological tests are currently the main diagnostic methods for SARS-CoV-2 (Ishige et al., 2020). As the golden standard, RT-PCR is highly reliable (Bustin & Nolan, 2020; Padoan et al., 2020). However, the implementation costs and relatively cumbersome operation problems make it a big challenge for large population screening (Thabet et al., 2020). The antibody-capture serological test is convenient, but seroconversion generally occurs in the second or third week of illness. Therefore, it is not ideal for the early diagnosis of infection (Hachim et al., 2020; Liu et al., 2020; Tang et al., 2020). The antigen-capture test is an alternative diagnostic method that relies on the immunodetection of viral antigens in clinical samples. Accordingly, this method could be applied for the detection of early infection no matter if the patient was asymptomatic or not (Ohnishi, 2008). Compared with RT-PCR based detection method, it is relatively inexpensive and can be used at the point-of-care.

Rapid viral antigen detection has been successfully used for diagnosing respiratory viruses such as influenza and respiratory syncytial viruses (Cazares et al., 2020; Ji et al., 2011; Ohnishi et al., 2005, 2012; Qiu et al., 2005). The sensitivity and specificity of the antigen-capture detection system depend highly on the antigen employed to generate antibodies (Ohnishi et al., 2012). The spike protein is one of the structural proteins of SARS-CoV-2, with the majority located on the outside surface of the viral particles (Fehr & Perlman, 2015; Kumar et al., 2020; Woo et al., 2005). It has a 76.4% homology with the spike protein of SARS-CoV. Sunwoo et al. (2013) showed that the bi-specific spike protein derived monoclonal antibody system exhibited excellent sensitivity in SARS-CoV detection. The virus infection is initiated by the interaction of spike protein receptor-binding domain (RBD) and angiotensin-converting enzyme 2 (ACE2) on host cells. It is widely accepted that the spike protein is one of the earliest antigenic proteins recognized by the host immune system (Callebaut, Enjuanes & Pensaert, 1996; Chen et al., 2020c; Gomez et al., 1998; Lu et al., 2004; Sanchez et al., 1999). Nevertheless, the difficulties of using spike protein as an antigen are also obvious. Firstly, it is not easy to express and purify the full-length spike protein (Tan et al., 2004). Besides, the spike protein is highly glycosylated (Kumar et al., 2020) and prone to mutation (Wang et al., 2020a), which may counteract the sensitivity of antigen-capture based detection method. Hence, it is critical to truncating the glycosylation and mutation sites on spike protein as much as possible in antigen design (Meyer, Drosten & Muller, 2014; Tan et al., 2004). A study using the truncated spike protein to detect SARS-CoV achieved a diagnostic sensitivity of >99% and a specificity of 100% (Mu et al., 2008), which suggests that the truncated spike protein of SARS-CoV-2 could also be an appropriate candidate for the early diagnostic testing and screening of SARS-CoV-2. In this study, we analyzed the spike protein via bioinformatics tools to obtain immunodominant fragments. The predicted sequences were joined together as a novel antigen for the immunization of mice and antibody production. Epitopes information presented by this work may aid in developing a promising antigen-capture based detection system in pandemic surveillance and containment.

## Method

### Data retrieval and sequence alignment

**Figure 1:**
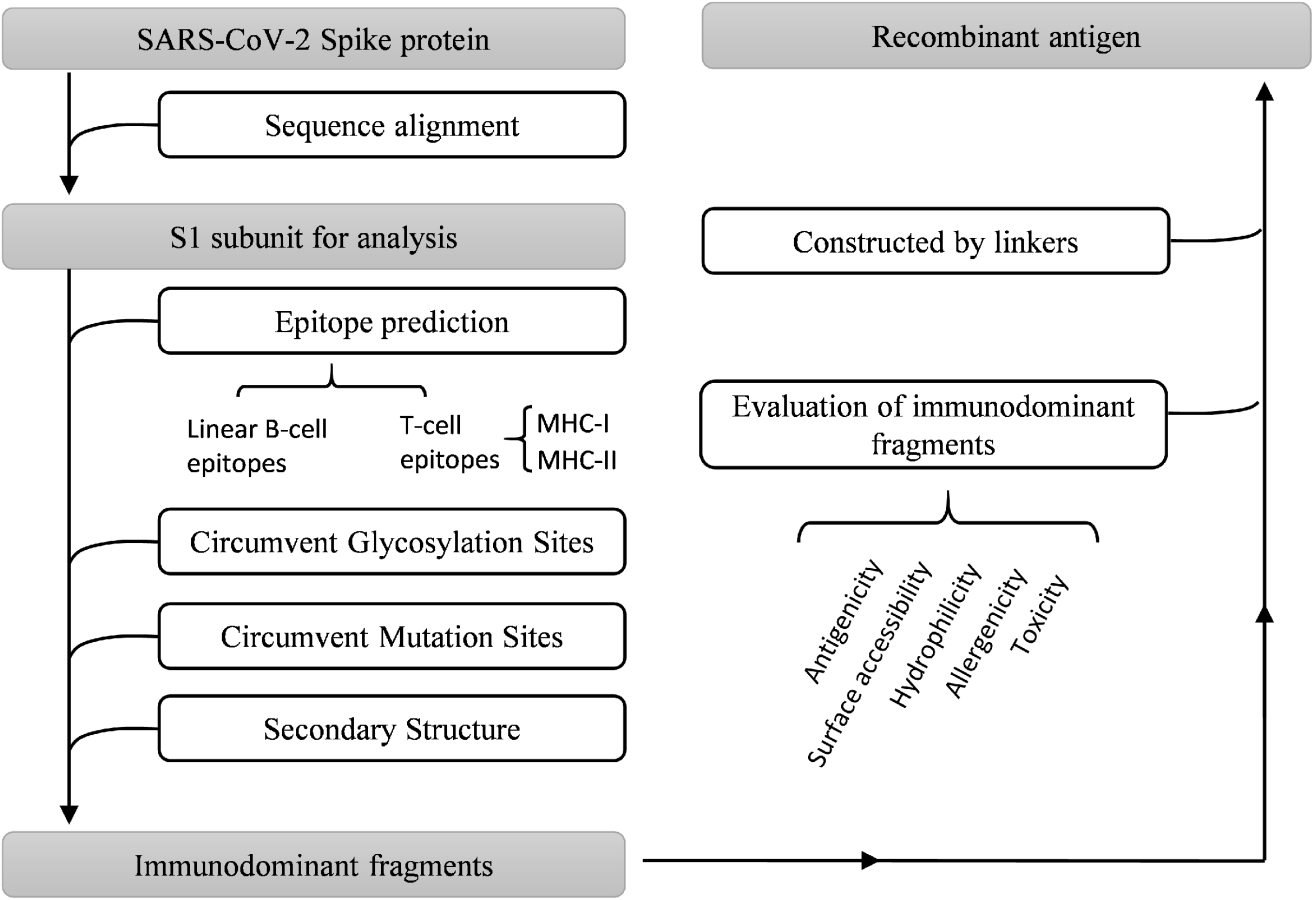
Work flow chart.

**Figure 2:**
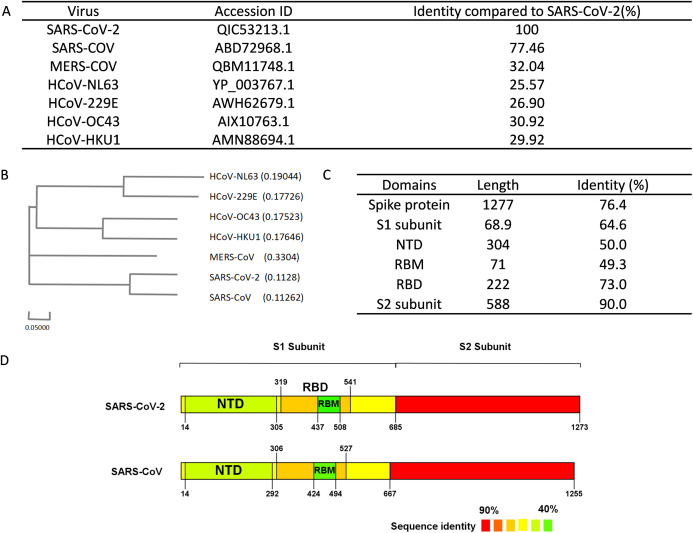
Sequence alignment results of spike protein. (A) Accession IDs and sequence identities of selected coronavirus spike protein. (B) Phylogenetic tree of spike proteins among selected coronavirus. (C) Sequence identity of major domains in spike protein between SARS-CoV-2 and SARS-CoV. (D) Sequence identity of domains in SARS-CoV-2 and SARS-CoV reflected by colors. From red to green, the color changing represents the sequence identity from high to low.

Multiple bioinformatics analysis tools were used in this study, and the flowchart is depicted in Fig. 1. Coronaviruses had four genera composed of alpha-, beta-, gamma- and delta-coronaviruses. Among them, alpha- and beta- genera could infect humans. Seven beta-coronaviruses are known to infect humans (HCoV-229E, HCoV-OC43, HCoV-NL63, HCoV-HKU1, SARS-CoV, MERS-CoV, and SARS-CoV-2) (Kin et al., 2015; Su et al., 2016). We utilized the NCBI database to obtain the sequences of these human-related coronaviruses spike proteins, of which accession numbers were presented in Fig. 2A. The Clustal Omega Server-Multiple Sequence Alignment was used to analyze the sequence similarity. The analysis of the phylogenetic tree was calculated by the same server. In this study, we set parameters of Clustal Omega as default (Sievers et al., 2011). Additionally, we conducted the EMBOSS Needle Server-Pairwise Sequence Alignment (Needleman & Wunsch, 1970) to compare the whole sequence and several major domains between SARS-CoV-2 and SARS-CoV to find out the specific genomic regions on SARS-CoV-2.

### Linear B-cell epitope prediction

Linear B-cell epitopes of the SARS-CoV-2 spike protein were calculated by ABCpred and Bepipred v2.0 servers. For ABCpred, we set a threshold of 0.8 to achieve a specificity of 95.50% and an accuracy of 65.37% for prediction. The window length was set to 16 (the default window length) in this study (Saha & Raghava, 2006). The BepiPred v2.0 combines a hidden Markov model and a propensity scale method. The score threshold for the BepiPred v2.0 was set to 0.5 (the default value) to obtain a specificity of 57.16% and a sensitivity of 58.56% (Jespersen et al., 2017). The residues with scores above 0.5 were predicted to be part of an epitope.

### T-cell epitope prediction

The free online service TepiTool server, integrated into the Immune Epitope Database (IEDB), was used to forecast epitopes binding to mice MHC molecules (Paul et al., 2016). Alleles including H-2-Db, H-2-Dd, H-2-Kb, H-2-Kd, H-2-Kk, and H-2-Ld were selected for MHC-I binding epitopes analysis. We checked the “IEDB recommended” option during computation and retained sequences with predicted consensus percentile rank ≤1 as predicted epitope (Trolle et al., 2015). For MHC-II binding epitopes, alleles including H2-IAb, H2-IAd, and H2-IEd were selected for analysis. As the same as MHC-I binding computation, we chose the “IEDB recommended” option, and peptides with predicted consensus percentile rank ≤10 were identified as potential epitopes (Wang et al., 2010; Zhang et al., 2012).

### Profiling and evaluation of selected fragments

The secondary structure of the SARS-CoV-2 spike protein (PDB ID: 6VSB chain B) was calculated by the PyMOL molecular graphics system using the SSP algorithm. PyMOL (http://www.pymol.org) is a python-based tool, which is widely used for visualization of macromolecules, such as SARS-CoV-2 spike protein in the current study (Yuan et al., 2016). Vaxijen2.0 server was utilized to analyze the antigenicity of epitopes and selected fragments. A default threshold of 0.4 was set and the prediction accuracy is between 70% and 89% (Doytchinova & Flower, 2007). The hydrophilicity of the selected fragment was analyzed by the online server ProtScale (Wilkins et al., 1999). Surface accessibility of predicted fragments was evaluated by NetsurfP, an online server calculating the surface accessibility and secondary structure of amino acid sequence (Petersen et al., 2009). Critical features such as allergenicity and toxicity were evaluated by online server AllerTOP v2.0 (Dimitrov et al., 2014) and ToxinPred (Gupta et al., 2013). In addition, we utilized IEDB (www.iedb.org) to search the selected fragments and epitopes to clarify whether these peptides have been experimentally verified (Vita et al., 2019). Protein sequence BLAST was performed to evaluate the possibility of cross-reactivity with other mouse protein sequences (Altschul et al., 1997).

## Results

### Sequence alignment of spike protein in different coronaviruses

We performed sequence alignment to determine the evolutionary relationships between SARS-CoV-2 and other beta-coronaviruses that could infect humans. According to the results of sequence alignment (Figs. 2A and 2B), SARS-CoV is the closest virus to SARS-CoV-2 among the seven HCoVs, exhibiting a 77.46% sequence identity. To better understand the divergence of spike protein sequences between SARS-CoV-2 and SARS-CoV, we further analyzed the sequences of main domains. Results showed that the S2 subunit was the most conserved domain with a 90.0% identity. RBM and NTD domains, which were located in the S1 subunit, exhibited 49.3% and 50.0% identity respectively (Figs. 2C and 2D). Hence, we chose the S1 subunit (amino acid 1-685) for the subsequent bioinformatics analysis given their high specificity.

### Linear B-cell epitope prediction of S1 subunit in SARS-CoV-2 spike protein

**Figure 3:**
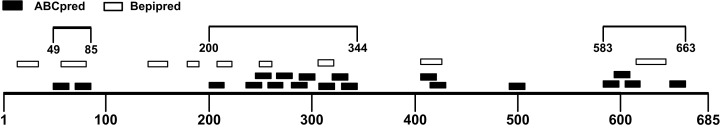
Preliminary immunodominant fragments based on B-cell epitope prediction results. The black squares represent epitopes predicted by ABCpred server, the black frames represent epitopes predicted by Bepipred v2.0 server, and the black lines with numbers on both ends represent the preliminary candidate immunodominant fragments.

The B-cell epitope is a surface accessible cluster of amino acids, which could be recognized by secreted antibodies or B-cell receptors and elicit humoral immune response (Getzoff et al., 1988). The immunodominant fragments should contain high-quality linear B-cell epitopes to stimulate antibody production effectively. The sequence of the SARS-CoV-2 S1 subunit was evaluated via ABCpred and BepiPred v2.0. A total of 31 peptides were identified by the ABCpred algorithm (Table S1). For the Bepipred v2.0 server, 14 epitopes were forecasted (Table S2). After antigenicity evaluation, 19 and 9 potential linear B-cell epitopes predicted by the ABCpred server and BepiPred v2.0 server were obtained respectively (Table 1). The peptides predicted by both bioinformatics programs are more likely to be an epitope recognized in vivo. After mapping the positions of peptides identified by these servers, 3 regions containing predicted epitopes were obtained. These regions could be preliminarily considered as candidates for immunodominant fragments (Fig. 3; Table 2).

**Table 1:** Linear B-cell epitopes predicted by ABCpred and BepiPred v2.0 with antigenicity score exceed the threshold value.

Tools	Position	Sequence	Length	Antigenicity (cut off ≥ 0.4)
ABCpred	583-598	EILDITPCSFGGVSVI	16	1.3971
	406-421	EVRQIAPGQTGKIADY	16	1.3837
	415-430	TGKIADYNYKLPDDFT	16	0.9642
	648-663	GCLIGAEHVNNSYECD	16	0.848
	288-303	AVDCALDPLSETKCTL	16	0.7905
	604-619	TSNQVAVLYQDVNCTE	16	0.7593
	307-322	TVEKGIYQTSNFRVQP	16	0.6733
	200-215	YFKIYSKHTPINLVRD	16	0.657
	257-272	GWTAGAAAYYVGYLQP	16	0.621
	329-344	FPNITNLCPFGEVFNA	16	0.6058
	245-260	HRSYLTPGDSSSGWTA	16	0.6017
	280-295	NENGTITDAVDCALDP	16	0.5804
	49-64	HSTQDLFLPFFSNVTW	16	0.5305
	492-507	LQSYGFQPTNGVGYQP	16	0.5258
	70-85	VSGTNGTKRFDNPVLP	16	0.5162
	236-251	TRFQTLLALHRSYLTP	16	0.5115
	266-281	YVGYLQPRTFLLKYNE	16	0.5108
	594-609	GVSVITPGTNTSNQVA	16	0.4651
	320-335	VQPTESIVRFPNITNL	16	0.4454
Bepipred v2.0	179-190	LEGKQGNFKNLR	12	1.1188
	404-426	GDEVRQIAPGQTGKIADYNYKLP	23	1.1017
	14-34	QCVNLTTRTQLPPAYTNSFTR	21	0.7594
	56-81	LPFFSNVTWFHAIHVSGTNGTKRFDN	26	0.6041
	208-222	TPINLVRDLPQGFSA	15	0.5531
	141-160	LGVYYHKNNKSWMESEFRVY	20	0.5308
	249-261	LTPGDSSSGWTAG	13	0.495
	306-321	FTVEKGIYQTSNFRVQ	16	0.4361
	615-644	VNCTEVPVAIHADQLTPTWRVYSTGSNVFQ	30	0.4259

**Table 2:** Details of epitopes in the preliminary immunodominant fragments selected according to linear B-cell epitope prediction results.

Regions	Epitope predicted by ABCpred			Epitope predicted by Bepipred v2.0		
	Position	Sequence	Antigenicity	Position	Sequence	Antigenicity
49-85	49-64	HSTQDLFLPFFSNVTW	0.5305	56-81	LPFFSNVTWFHAIHVSGTNGTKRFDN	0.6041
	70-85	VSGTNGTKRFDNPVLP	0.5162			
200-344	200-215	YFKIYSKHTPINLVRD	0.6570	208-222	TPINLVRDLPQGFSA	0.5531
	236-251	TRFQTLLALHRSYLTP	0.5115	249-261	LTPGDSSSGWTAG	0.4950
	245-260	HRSYLTPGDSSSGWTA	0.6017			
	257-272	GWTAGAAAYYVGYLQP	0.6210			
	266-281	YVGYLQPRTFLLKYNE	0.5108			
	280-295	NENGTITDAVDCALDP	0.5804			
	288-303	AVDCALDPLSETKCTL	0.7905	306-321	FTVEKGIYQTSNFRVQ	0.4361
	307-322	TVEKGIYQTSNFRVQP	0.6733			
	320-335	VQPTESIVRFPNITNL	0.4454			
	329-344	FPNITNLCPFGEVFNA	0.6058			
	415-430	TGKIADYNYKLPDDFT	0.9642			
583-663	583-598	EILDITPCSFGGVSVI	1.3971	615-644	VNCTEVPVAIHADQLTPTWRVYSTGSNVFQ	0.4259
	594-609	GVSVITPGTNTSNQVA	0.4651			
	604-619	TSNQVAVLYQDVNCTE	0.7593			
	648-663	GCLIGAEHVNNSYECD	0.8480			

### Murine T-cell epitope prediction of S1 subunit in SARS-CoV-2 spike protein

**Figure 4:**
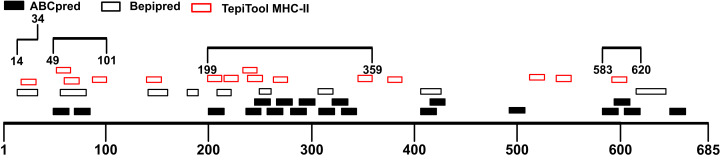
Adjusted candidate immunodominant fragments according to MHC-II T-cell epitope prediction results. The black squares represent epitopes predicted by ABCpred server, and the black frames represent epitopes predicted by Bepipred v2.0 server. The red frames denote MHC-II binding epitopes. The black lines with numbers on both ends represent the adjusted candidate fragments.

Though B cells are responsible for producing antibodies, humoral immunity is heavily dependent on the activation of T cells (Cho et al., 2019a). Helper T cells (Th) recognize antigen peptides presented by MHC-II molecules and facilitate the humoral immune response (Cho et al., 2019b; Mahon et al., 1995). During humoral immune responses, antigen-activated T cells could provide help in many aspects including directing antibody class switching and guiding the differentiation of antibody-secreting plasma cells as well as the properties of the B-cell antigen receptor (Cho et al., 2019a; Paus et al., 2006; Shulman et al., 2014). Therefore, the immunodominant fragments containing T-cell epitopes could offer essential help to powerful antibody production. The S1 subunit was selected for the prediction of T-cell epitopes. We utilized the TepiTool server to forecast MHC-I and MHC-II binding epitopes. A total of 35 MHC-I binding epitopes was predicted (Table S3), and 27 peptides were identified as MHC-II binding epitopes (Table S4). The antigenicity of these peptides was calculated via Vaxijen 2.0 server (Table 3). Combined with the MHC-II epitopes prediction results, the candidate immunodominant fragments were adjusted (Fig. 4). Compared with the preliminary candidate immunodominant fragments screened according to the linear B-cell epitope prediction, we added the Spike_14-34_ fragment into consideration because it contains a linear B epitope and an MHC-II binding epitope, both of which had high antigenicity scores (Table 4).

**Table 3:** MHC-II and MHC-I binding epitopes predicted by TepiTool server with antigenicity score exceed threshold value.

Type	Position	Sequence	Length	Allele	Core (smm-align)	Core (nn-align)	Percentile Rank	Antigenicity (cut off ≥ 0.4
MHC-II binding	538-552	CVNFNFNGLTGTGVL	15	H2-IAb	FNFNGLTGT	FNFNGLTGT	8.55	1.3281
	374-388	FSTFKCYGVSPTKLN	15	H2-IAb	FKCYGVSPT	YGVSPTKLN	6.45	1.0042
	199-213	GYFKIYSKHTPINLV	15	H2-Iab	KIYSKHTPI	YSKHTPINL	6.9	0.9278
	18-32	LTTRTQLPPAYTNSF	15	H2-IAb	TRTQLPPAY	TRTQLPPAY	9.9	0.79
	60-74	SNVTWFHAIHVSGTN	15	H2-IAb	VTWFHAIHV	TWFHAIHVS	9.1	0.7044
	263-277	AAYYVGYLQPRTFLL	15	H2-IAb	VGYLQPRTF	VGYLQPRTF	8.75	0.6073
	592-606	FGGVSVITPGTNTSN	15	H2-IAb	VITPGTNTS	VSVITPGTN	6	0.5825
	238-252	FQTLLALHRSYLTPG	15	H2-IEd	TLLALHRSY	TLLALHRSY	9.85	0.5789
	345-359	TRFASVYAWNRKRIS	15	H2-IAb	FASVYAWNR	YAWNRKRIS	7.45	0.4963
	215-229	DLPQGFSALEPLVDL	15	H2-IAb	FSALEPLVD	FSALEPLVD	6.05	0.4812
	140-154	FLGVYYHKNNKSWME	15	H2-IEd	GVYYHKNNK	YYHKNNKSW	6.4	0.4793
	512-526	VLSFELLHAPATVCG	15	H2-IAb	FELLHAPAT	FELLHAPAT	2.9	0.4784
	87-101	NDGVYFASTEKSNII	15	H2-Iab	YFASTEKSN	VYFASTEKS	6.85	0.4277
	52-66	QDLFLPFFSNVTWFH	15	H2-IAb	FLPFFSNVT	FLPFFSNVT	2.95	0.4159
	233-247	INITRFQTLLALHRS	15	H2-IAd	ITRFQTLLA	ITRFQTLLA	1.9	0.4118
MHC-I binding	643-651	FQTRAGCLI	9	H-2-Kk			0.6	1.7332
	612-620	YQDVNCTEV	9	H-2-Db			0.4	1.6172
	539-547	VNFNFNGLT	9	H-2-Kb			0.47	1.5069
	503-511	VGYQPYRVV	9	H-2-Kb			0.47	1.4383
	379-387	CYGVSPTKL	9	H-2-Kd			0.3	1.4263
	16-24	VNLTTRTQL	9	H-2-Kb			0.86	1.3468
	510-518	VVVLSFELL	9	H-2-Kb			0.43	1.0909
	202-210	KIYSKHTPI	9	H-2-Kb			0.27	0.7455
	168-176	FEYVSQPFL	9	H-2-Kk			0.5	0.6324
	268-276	GYLQPRTFL	9	H-2-Kd			0.2	0.6082
	505-513	YQPYRVVVL	9	H-2-Dd			0.3	0.5964
	488-496	CYFPLQSYG	9	H-2-Kd			0.64	0.578
	215-223	DLPQGFSAL	9	H-2-Dd			0.69	0.5622
	342-350	FNATRFASV	9	H-2-Kb			0.56	0.5609
	84-92	LPFNDGVYF	9	H-2-Ld			0.21	0.5593
	484-492	EGFNCYFPL	9	H-2-Kb			0.84	0.5453
	62-70	VTWFHAIHV	9	H-2-Kb			0.61	0.5426
	489-497	YFPLQSYGF	9	H-2-Dd			0.8	0.5107
	350-358	VYAWNRKRI	9	H-2-Kd			0.7	0.5003
	60-68	SNVTWFHAI	9	H-2-Kb			0.82	0.4892
	262-270	AAAYYVGYL	9	H-2-Kb			0.98	0.4605

**Table 4:** Details of candidate immunodominant fragments adjusted according to the MHC-II binding T-cell epitopes prediction results.

Regions	Linear B-cell epitopes				MHC-II binding epitopes		
	Tools	Position	Sequence	Antigenicity	Position	Sequence	Antigenicity
14-34	Bepipred v2.0	14-34	QCVNLTTRTQLPPAYTNSFTR	0.7594	18-32	LTTRTQLPPAYTNSF	0.7900
49-101	Bepipred v2.0	56-81	LPFFSNVTWFHAIHVSGTNGTKRFDN	0.6041	52-66	QDLFLPFFSNVTWFH	0.4159
	ABCpred	49-64	HSTQDLFLPFFSNVTW	0.5305	60-74	SNVTWFHAIHVSGTN	0.7044
	ABCpred	70-85	VSGTNGTKRFDNPVLP	0.5162	87-101	NDGVYFASTEKSNII	0.4277
199-359	Bepipred v2.0	208-222	TPINLVRDLPQGFSA	0.5531	199-213	GYFKIYSKHTPINLV	0.9278
		249-261	LTPGDSSSGWTAG	0.4950			
		306-321	FTVEKGIYQTSNFRVQ	0.4361			
	ABCpred	200-215	YFKIYSKHTPINLVRD	0.6570			
		236-251	TRFQTLLALHRSYLTP	0.5115	215-229	DLPQGFSALEPLVDL	0.4812
		245-260	HRSYLTPGDSSSGWTA	0.6017			
		257-272	GWTAGAAAYYVGYLQP	0.6210	233-247	INITRFQTLLALHRS	0.4118
		266-281	YVGYLQPRTFLLKYNE	0.5108			
		280-295	NENGTITDAVDCALDP	0.5804	238-252	FQTLLALHRSYLTPG	0.5789
		288-303	AVDCALDPLSETKCTL	0.7905			
		307-322	TVEKGIYQTSNFRVQP	0.6733	263-277	AAYYVGYLQPRTFLL	0.6073
		320-335	VQPTESIVRFPNITNL	0.4454			
		329-344	FPNITNLCPFGEVFNA	0.6058			
					345-359	TRFASVYAWNRKRIS	0.4963
583-620	ABCpred	583-598	EILDITPCSFGGVSVI	1.3971	592-606	FGGVSVITPGTNTSN	0.5825
		594-609	GVSVITPGTNTSNQVA	0.4651			
		604-619	TSNQVAVLYQDVNCTE	0.7593			

### Immunodominant fragments refinement according to the glycosylation site distribution, mutation site distribution, and secondary structure

A profile of 24 glycosylation sites of SARS-CoV-2 spike protein has been reported (Shajahan et al., 2020). Since glycans could hinder the recognition of antigens by shielding the residues (Walls et al., 2019), protein glycosylation would affect the performance of antigen detection. Thus, glycosylation sites should be circumvented when selecting the immunodominant fragments. According to the study of Shajahan et al. (2020), 15 glycosylation sites were located in the S1 subunit of the spike protein. Hence, the fragments in this study were adjusted to Spike_14-34_, Spike_49-101_, Spike_199-261_, and Spike_583-620_. To retain antigenicity of the epitopes, the final identified fragments only contained 3 glycosylation sites which should have a minimum effect on antigen recognition.

Rapid transmission of COVID-19 provides the SARS-CoV-2 with substantial opportunities for natural selection and mutations. To ensure the stability of the detection method, the immunodominant fragments were modified to avoid high-frequency mutation sites (Wang et al., 2020b). Spike_14-34_ were excluded for containing four high-frequency mutation sites. Fragment Spike_49-101_ was adjusted to Spike_56-92_, and fragment Spike_583-620_ was adjusted to Spike_583-609_. By adjusting the fragments, we avoided in a total of 8 high-frequency mutation sites (L5F, L18F, T29I, R21K/T, H49Y, L54F, S98F, D614G). The mainly mutant sites on the recent emergent highly infectious variants (including B.1.1.7, B.1.351, and P.1), such as N501Y, D614G, E484K, Y144del, K417N, and A570D were also not included in our fragments. The adjusted fragments contain none of the above high-frequency mutation sites, which might avoid the impact of mutations on detection performance and improve the detection efficiency in the future (Li et al., 2020; Tegally et al., 2020).

**Figure 5:**
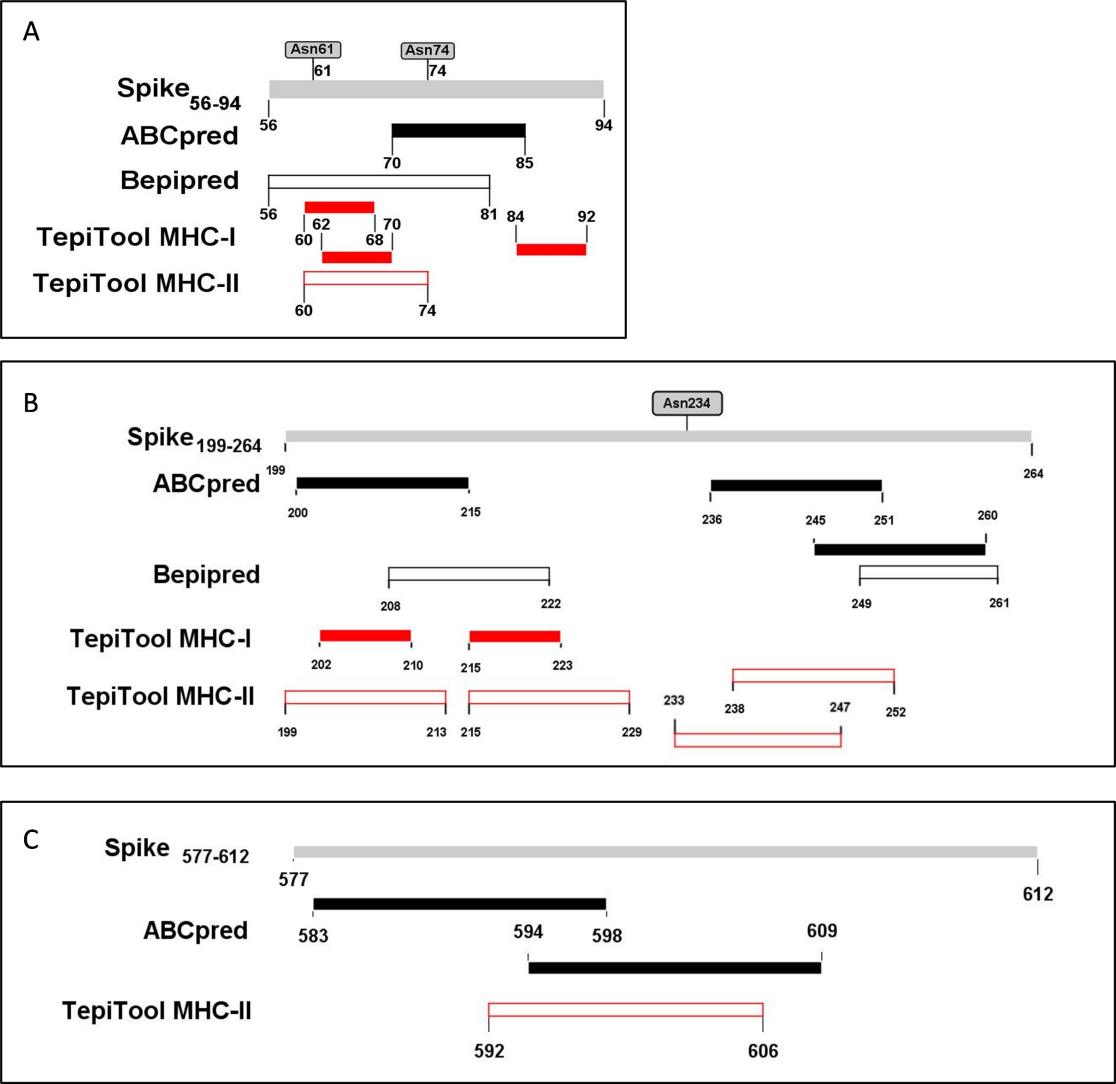
The epitopes and glycosylation sites on the selected immunodominant fragments. (A–C) Present the predicted epitopes and glycosylation sites on fragment Spike56-94, Spike199-264 and Spike577-612 respectively. The black squares represent epitopes predicted by ABCpred server, and the black frames represent epitopes predicted by Bepipred v2.0 server. The red squares represent MHC-I binding epitopes, and the red frames represent MHC-II binding epitopes. The gray squares mean occupied glycosylation sites contained in the selected fragments.

The PyMOL was used to present the secondary structure of the spike protein (PDB ID: 6VSB) (Fig. S1). To keep the integrity of the secondary structure of the selected fragments, we extended the N- and C-ends with 2~5 residues, and the immunodominant fragments were finally adjusted to Spike_56-94_, Spike_199-264_, and Spike_577-612._ The epitopes and potential glycosylation sites contained in the selected immunodominant fragments were displayed in Fig. 5.

### Profiling, evaluation, and visualization of selected immunodominant fragments

To further evaluate the antibody binding potentiality of these antigenic regions, the key features of the selected fragments such as antigenicity, hydrophilicity, surface accessibility, toxicity, and allergenicity were analyzed and presented (Table 5). The hydrophilicity and surface accessibility of the spike protein subunit 1 were calculated. The selected fragments of interest were submitted for computation of antigenicity, toxicity, and allergenicity. Three fragments presented relatively moderate hydrophilicity and surface accessibility. The proportion of hydrophilic amino acids in the selected fragments Spike_56-94_, Spike_199-264_, Spike_577-612_ are 48.72%, 45.45%, 33.33% respectively. The surface accessibility of these fragments calculated by the online server was shown in Table 5.

**Table 5:** Significant features of the selected immunodominant fragments. The sequences marked as bold and italic in the table represent amino acids with hydrophilicity and surface accessibility respectively.

Fragments	Spike_56-94_	Spike_199-264_	Spike_577-612_
Length(aa)	39	66	36
Sequence	LPFFSNVTWFHAIHVSGTNGTKRFDNPVLPFNDGVYFAS	GYFKIYSKHTPINLVRDLPQGFSALEPLVDLPIGINITRFQTLLALHRSYLTPGDSSSGWTAGAAA	RDPQTLEILDITPCSFGGVSVITPGTNTSNQVAVLY
Antigenicity	0.4590	0.5774	0.9127
Domain	S1(NTD)	S1(NTD)	S1
Hydrophilicity fragments	LPFFSNVTWFHAIH***V***S***GTNGTKRFDNPVLP***FNDGV***YFAS***	***GY***F***KIYSKHTPIN***LV***RD***L***PQ***GF***S***ALEPLVDLPIGINIT***R***FQTLLALHRS***YLTPGDSSSGWT***AGAAA	***RDPQTL***EILDITPCSFGGVSVITPG***TNTSN***QVA***V***LY
Surface Accessibility fragments	LP***FFSN***VTWFHAI***H***V***SGTNGTKR***F***D***N***P***VL***P***F***ND***GVYFAS	G***Y***FKIYSK ***HTPINLVRD***L***PQGF***SAL***E***PLV***D***LPIGI***N***I***TR***FQTLLALHRS***YLTPGDSSS***GWTAGAAA	***R***D***PQT***L***E***IL***D***I***T***PC***SFG***GVSVIT***PGTNTSNQ***VAVLY
Toxicity	Non-toxin	Non-toxin	Non-toxin
Allergenicity	non-allergen	non-allergen	probable allergen

**Figure 6:**
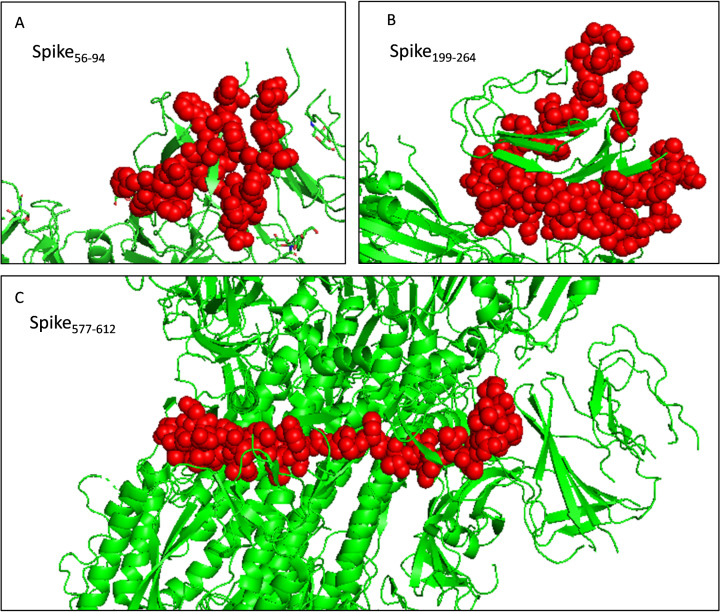
Selected immunodominant fragments presented as spheres in the trimer structure of spike protein viewed by PyMOL. Selected fragments were presented as red spheres, green cartoons denote unselected sections. (A, B, and C) denote fragments Spike_56-94_, Spike_199-264_, and Spike_577-612_ respectively.

The toxicity of the selected fragments was examined and no fragment was predicted to be toxic. The allergenicity was assessed and only fragment Spike_577-612_ was predicted to be a probable allergen. Attention should be paid to monitor potential allergic reactions when injecting the recombinant protein into mice. And the selected fragments were presented as the sphere in the trimer structure (Fig. 6). Next, we scanned the selected fragments utilizing the IEDB database to determine whether they were experimentally tested. The results showed that Spike_200-215_(IEDB ID: 1330367) and Spike_238-252_ (IEDB ID: 1329417) were identified experimentally as HLA class II epitope in SARS-CoV-2. Spike84-92 (IEDB ID: 1321049) and Spike202-210 (IEDB ID: 1319559) have been experimentally proved as HLA-B epitopes (Table S5). These findings enhanced the credibility of the current in silico analysis. The fragments identified would have a strong capacity in stimulating powerful antibody production.

### Immunodominant fragments based recombinant antigen design

**Figure 7:**
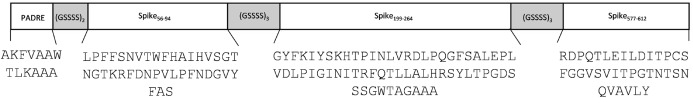
A schematic diagram of recombinant peptide composed of selected fragments and a PADRE epitope.

Three immunodominant fragments embody several linear B-cell epitopes, MHC-I binding, and MHC-II binding T-cell epitopes were selected. As a universal Th epitope, the PAN DR epitope [PADRE(AKFVAAWTLKAAA)] was added into the construction aiming to boost helper T cell activity (Alexander et al., 2000; Ghaffari-Nazari et al., 2015). (GGGGS)_n_ is a wildly used flexible linker with the function of segmenting protein fragments, maintaining protein conformation, preserving biological activity, and promoting protein expression (Chen, Zaro & Shen, 2013). Finally, we combined the fragments and a PADRE epitope by linker peptide (GGGGS)_2_ and (GGGGS)_3_ (Chen, Zaro & Shen, 2013) (Fig. 7). The predicted antigenicity of the final construct (194 aa) was 0.5690 (Table 6). A protein BLAST for the final construct was conducted to evaluate the possibility of cross-reactivity. The BLAST result suggested that, except for the SARS-CoV-2 spike protein, no protein would cross-react with the construct (Raw data in the Supplemental Files), which indicated that our fragments possess good specificity.

**Table 6:** The structure and antigenicity of final recombinant peptides.

Final construct	PAN DR + (GGGGS)_2_ + Spike_56-94_ +(GGGGS)_3_ + Spike_199-264_ +(GGGGS)_3_ + Spike_577-612_
Sequence	AKFVAAWTLKAAAGGGGSGGGGSLPFFSNVTWFHAIHVSGTNGTKRFDNPVLPFNDGVYFASGGGGSGGGGSGGGGSGYFKIYSKHTPINLVRDLPQGFSALEPLVDLPIGINITRFQTLLALHRSYLTPGDSSSGWTAGAAAGGGGSGGGGSGGGGSRDPQTLEILDITPCSFGGVSVITPGTNTSNQVAVLY
Antigenicity	0.5690

## Discussion

In this study, the immunodominant fragments within the S1 subunit of the SARS-CoV-2 spike protein were explored. The final construct consists of three immunodominant fragments Spike_56-94_, Spike_199-264_, Spike_577-612_, and a PADRE epitope. The recombinant antigen will be used to immunize mice to generate qualified antibody which could be applied for developing an antigen-capture based detection system.

The antibody-based antigen capturing method is user-friendly, time-saving, and economical. Thus, it is an ideal complementary detection strategy especially for early diagnosis and large population screening. The monoclonal antibodies against SARS-CoV have been successfully applied in the immunological antigen-detection of SARS-CoV (Ohnishi, 2008). Accordingly, we explored the immunodominant fragments on the spike protein of SARS-CoV-2, which would provide aid in developing an accurate and fast antigen-capture based early detection system for SARS-CoV-2.

We selected the S1 subunit for immunodominant fragments screening after divergence analysis. It had been reported that an S1 antigen-based assay of SARS-CoV could capture the virus as soon as the infection occurs (Sunwoo et al., 2013). Jong-Hwan Lee et al. designed a method that could seize and detect spike protein S1 subunit of SARS-CoV-2 using ACE2 receptor and S1-mAb (Lee et al., 2021). This finding suggests that it is appropriate to use the S1 subunit for specific and early diagnosis of SARS-CoV-2. Three immunodominant fragments (Spike_56-94_, Spike_199-264_, and Spike_577-612_) were identified in the present study. These sequences will be joined to construct recombinant peptides in the next step. Instead of using inactivated full-length spike protein, we designed a novel recombinant protein construct that increased sequence specificity as well as circumvented mutation sites and glycosylation sites. As the antigen design is based on bioinformatics study, the exact ability of the selected fragments to produce qualified antibodies for virus detection has yet to be determined by experiments.

Noticeably, the spike protein of SARS-CoV-2 is heavily glycosylated. Glycans could shield epitopes during antibody recognition, which may interfere with the detection of viral proteins (Shajahan et al., 2020). About 17 N-glycosylation sites along with two O-glycosylation sites were found occupied in the spike protein of SARS-CoV-2 (Shajahan et al., 2020). We circumnavigated most glycosylation sites when selecting immunodominant fragments. The three selected fragments in this study only contain 3 glycosylation sites. In case these glycosylation sites impede the diagnostic performance, an additional deglycosylation step with N-glycanase should be applied for the test specimens (Dermani et al., 2019), which is a simple and efficient method for deglycosylation (Hirani, Bernasconi & Rasmussen, 1987; Huang et al., 2015; Lattová et al., 2016; Zheng, Bantog & Bayer, 2011). Alternatively, an eukaryotic expressing system could be employed to mimic the antigen presented in human cells.

Though coronaviruses can find and repair errors during the replication process (Wang et al., 2020b), the SARS-CoV-2 genome still presents a large number of mutations. Mutations could not only help virus slip past our immune defense, but also spoil the efficiency of diagnostic tests (Chen et al., 2020b). In this study, we circumvented high-frequency mutation sites when selecting antigen fragments. In addition, our fragments also avoided RBD regions which are prone to mutation (Chen et al., 2020b). The construct finally built contained no high-frequency mutation.

To date, several studies using predictive algorithms to analyze SARS-CoV-2 have been reported (Alam et al., 2020; Behmard et al., 2020; Can et al., 2020; Chen et al., 2020a; Dong et al., 2020; Poran et al., 2020; Saha, Ghosh & Burra, 2021; Sohail et al., 2021). However, most of these bioinformatics analyses against SARS-CoV-2 intended to develop effective vaccines to prevent infection and the identified sequences possess high homology with other viruses, especially SARS-CoV (Bhattacharya et al., 2020; Chen et al., 2020a; Robson, 2020). On the contrary, the fragments suitable for diagnosis should be unique when compared with other species to ensure the specificity of detection. Therefore, the results obtained from vaccine studies are not ideal for virus detection. In this study, attention was paid to the sequences with high variability, hence the immunodominant fragments identified are more specific. Distinct from vaccine studies, murine MHC alleles were selected in epitopes prediction in this study, so that the designed antigen could trigger a strong humoral immune response in mice. Furthermore, glycosylated sites and recently identified high-frequency mutation sites were deliberately avoided during the screening process to eliminate their potential adverse impact.

In silico analysis has been widely used to mine and identify various pathogens as well as epitopes prediction (Kiyotani et al., 2020; Liò & Goldman, 2004; Qin et al., 2003; Robson, 2020; Shen et al., 2003). In this study, identified fragments were further scanned in the IEDB database, and found four peptides contained in the sequences were experimentally validated epitopes (Table S5), which reinforced the conclusion of the present study. In the following studies, we will immunize Balb/c mice with the designed antigen to generate mAbs which could be utilized for SARS-CoV-2 diagnosis after evaluating their sensitivity, specificity, and other related properties.

## Conclusion

Through bioinformatics analysis, three immunodominant fragments were identified in the present study. After connected by flexible linkers, we acquired a final recombinant peptide with 194 residues. It was predicted to possess high antigenicity and specificity for SARS-CoV-2. Our next move is to express and purify the recombinant protein in a suitable expression system, followed by immunizing the mice with purified immunogen to obtain specific antibodies. The present study would provide aid in developing an antigen-capture based detection system.

## Supplemental Information

10.7717/peerj.11232/supp-1 Supplemental Information 1 The secondary structure presentation of SARS-CoV2 spike using PyMOL server. The letter L with green shades represents the loop structure, the letter S with yellow shades denotes the sheet structure and the letter H with gray shades indicates the helix structure. The lines “-” represent gaps in the sequence when we use PyMOL to visually present it.

10.7717/peerj.11232/supp-2 Supplemental Information 2 Linear B-cell epitopes predicted by the ABCpred server along with their position, sequence, ABCpred prediction score and antigenicity score.

10.7717/peerj.11232/supp-3 Supplemental Information 3 Linear B-cell epitopes predicted by the Bepipred v2.0 server along with their position, sequence and antigenicity score.

10.7717/peerj.11232/supp-4 Supplemental Information 4 MHC-I binding epitopes predicted by TepiTool along with their position, sequence, allele and antigenicity score.

10.7717/peerj.11232/supp-5 Supplemental Information 5 MHC-II binding epitopes predicted by TepiTool along with their position, sequence, allele and antigenicity score.

10.7717/peerj.11232/supp-6 Supplemental Information 6 Experimentally verified epitopes of SARS-CoV-2 contained in the immunodominant fragments.

10.7717/peerj.11232/supp-7 Supplemental Information 7 Raw data. All prediction results including linear B-cell epitope, T-cell epitope, hydrophilicity and surface accessibility of SARS-CoV-2 spike protein S1 subunit.

## Additional Information and Declarations

### Data Availability

The following information was supplied regarding data availability: The raw measurements are available in the Supplemental File.
