# Supplementary material for: Bioinformatic prediction of immunodominant regions in spike protein for early diagnosis of the severe acute respiratory syndrome coronavirus 2 (SARS-CoV-2)
Source: PeerJ. 2021 Apr 8;9:e11232. doi: 10.7717/peerj.11232 (PMC8038641; doi:10.7717/peerj.11232)
Supplement: Supplemental Information 1 — The letter L with green shades represents the loop structure, the letter S with yellow shades denotes the sheet structure and the letter H with gray shades indicates the helix structure. The lines “-” represent gaps in the sequence when we use PyMOL to visually present it. [file peerj-09-11232-s001.pdf]

```
1        10        20        30        40        50
|        |         |         |         |         |
MFVFLVLLPLVSSQCVNLTTRTQLPPAYTNSFTRGVYYPDKVFRSSVLHS
--------------------------LSSSSLLLLLLLLLLLLLLLSSSS
TQDLFLPFFSNVTWFHAIHVSGTNGTKRFDNPVLPFNDGVYFASTEKSNI
SSSSSLLLLSSSSSSL--------------LLLLLLLLLSSSSSLLLLLL
IRGWIFGTTLDSKTQSLLIVNNATNVVIKVCEFQFCNDPFLGVYYHKNNK
LLLLLSSLLLLLLLLSSLLSSLLLLSSSSLLLLLLLLLLLL---------
SWMESEFRVYSSANNCTFEYVSQPFLMDLEGKQGNFKNLREFVFKNIDGY
----LLLLLLLLLLLLLLSSSSLLLL----------LLSSSSSSSSSLLS
FKIYSKHTPINLVRDLPQGFSALEPLVDLPIGINITRFQTLLALHRSYLT
SSSSSSSSL-------LLLLLLLSSSSSSLLLLLLLLLLLLL--------
PGDSSSGWTAGAAAYYVGYLQPRTFLLKYNENGTITDAVDCALDPLSETK
------------LLSSSLLLSSSSSSSSSLLLLLSSSSSSLLLLLHHHHH
CTLKSFTVEKGIYQTSNFRVQPTESIVRFPNITNLCPFGEVFNATRFASV
HHHHLLLLLLSSSSSSSSSLLLSSSSSSLLLLLLLLLHHHHHHLLLLLLL
YAWNRKRISNCVADYSVLYNSASFSTFKCYGVSPTKLNDLCFTNVYADSF
LLLSSSSSLLSSLLHHHHHHLLLLLSSSSLLLHHHHHHHHLLLSSSSSSS
VIRGDEVRQIAPGQTGKIADYNYKLPDDFTGCVIAWNSNNLDSKVGGNYN
SSSLHHHHHHLLLLLHHHHHHHLLLLLLLLLSSSSSSLLLLLL-----LL
YLYRLFRKSNLKPFERDISTEIYQAGSTPCNGVEGFNCYFPLQSYGFQPT
SSSL-----LLLLLLLLLLLLLL-------------LLLLLLSSSLLLLL
NGVGYQPYRVVVLSFELLHAPATVCGPKKSTNLVKNKCVNFNFNGLTGTG
--LLLLSSSSSSSSSSLLLLLLLSSLLLLLLLLLLLLSSSSSSLLLSSSS
VLTESNKKFLPFQQFGRDIADTTDAVRDPQTLEILDITPCSFGGVSVITP
SSSSLLLLLLLLLLSSSLLLLLSSSSSLLLLLLSSSSSLLLLLLLLLLLL
GTNTSNQVAVLYQDVNCTEVPVAIHADQLTPTWRVYSTGSNVFQTRAGCL
LLLLLLLLSSSSSLLLLLLL-----------------LLLLSSSSLLSSS
IGAEHVNNSYECDIPIGAGICASYQTQTNSPRRAR
SLLSSLLLLLLLLLSSSLLSSL-------------
```
