# Supplementary material for: Bioinformatic prediction of immunodominant regions in spike protein for early diagnosis of the severe acute respiratory syndrome coronavirus 2 (SARS-CoV-2)
Source: PeerJ. 2021 Apr 8;9:e11232. doi: 10.7717/peerj.11232 (PMC8038641; doi:10.7717/peerj.11232)
Supplement: Supplemental Information 2 [file peerj-09-11232-s002.docx]

| Position | Sequence | Score | Antigenicity |
| --- | --- | --- | --- |
| 583-598 | EILDITPCSFGGVSVI | 0.8 | 1.3971 |
| 406-421 | EVRQIAPGQTGKIADY | 0.85 | 1.3837 |
| 415-430 | TGKIADYNYKLPDDFT | 0.84 | 0.9642 |
| 648-663 | GCLIGAEHVNNSYECD | 0.9 | 0.848 |
| 288-303 | AVDCALDPLSETKCTL | 0.8 | 0.7905 |
| 604-619 | TSNQVAVLYQDVNCTE | 0.83 | 0.7593 |
| 307-322 | TVEKGIYQTSNFRVQP | 0.91 | 0.6733 |
| 200-215 | YFKIYSKHTPINLVRD | 0.84 | 0.657 |
| 257-272 | GWTAGAAAYYVGYLQP | 0.95 | 0.621 |
| 329-344 | FPNITNLCPFGEVFNA | 0.82 | 0.6058 |
| 245-260 | HRSYLTPGDSSSGWTA | 0.92 | 0.6017 |
| 280-295 | NENGTITDAVDCALDP | 0.83 | 0.5804 |
| 49-64 | HSTQDLFLPFFSNVTW | 0.8 | 0.5305 |
| 492-507 | LQSYGFQPTNGVGYQP | 0.9 | 0.5258 |
| 70-85 | VSGTNGTKRFDNPVLP | 0.82 | 0.5162 |
| 236-251 | TRFQTLLALHRSYLTP | 0.89 | 0.5115 |
| 266-281 | YVGYLQPRTFLLKYNE | 0.86 | 0.5108 |
| 594-609 | GVSVITPGTNTSNQVA | 0.95 | 0.4651 |
| 320-335 | VQPTESIVRFPNITNL | 0.8 | 0.4454 |
| 194-209 | FKNIDGYFKIYSKHTP | 0.8 | 0.3569 |
| 347-362 | FASVYAWNRKRISNCV | 0.81 | 0.3371 |
| 476-491 | GSTPCNGVEGFNCYFP | 0.91 | 0.2489 |
| 525-540 | CGPKKSTNLVKNKCVN | 0.86 | 0.2006 |
| 630-645 | TPTWRVYSTGSNVFQT | 0.84 | 0.1876 |
| 151-166 | SWMESEFRVYSSANNC | 0.86 | 0.1724 |
| 391-406 | CFTNVYADSFVIRGDE | 0.85 | 0.0861 |
| 564-579 | QFGRDIADTTDAVRDP | 0.82 | 0.0689 |
| 470-485 | TEIYQAGSTPCNGVEG | 0.89 | -0.0105 |
| 464-479 | FERDISTEIYQAGSTP | 0.86 | -0.2904 |
| 124-139 | TNVVIKVCEFQFCNDP | 0.8 | -0.3969 |
| 97-112 | KSNIIRGWIFGTTLDS | 0.84 | -0.5366 |
