# Supplementary material for: Bioinformatic prediction of immunodominant regions in spike protein for early diagnosis of the severe acute respiratory syndrome coronavirus 2 (SARS-CoV-2)
Source: PeerJ. 2021 Apr 8;9:e11232. doi: 10.7717/peerj.11232 (PMC8038641; doi:10.7717/peerj.11232)
Supplement: Supplemental Information 3 [file peerj-09-11232-s003.docx]

| Position | Sequence | Antigenicity |
| --- | --- | --- |
| 179-190 | LEGKQGNFKNLR | 1.1188 |
| 404-426 | GDEVRQIAPGQTGKIADYNYKLP | 1.1017 |
| 14-34 | QCVNLTTRTQLPPAYTNSFTR | 0.7594 |
| 56-81 | LPFFSNVTWFHAIHVSGTNGTKRFDN | 0.6041 |
| 208-222 | TPINLVRDLPQGFSA | 0.5531 |
| 141-160 | LGVYYHKNNKSWMESEFRVY | 0.5308 |
| 249-261 | LTPGDSSSGWTAG | 0.495 |
| 306-321 | FTVEKGIYQTSNFRVQ | 0.4361 |
| 65-644 | VNCTEVPVAIHADQLTPTWRVYSTGSNVFQ | 0.4259 |
| 440-493 | NLDSKVGGNYNYLYRLFRKSNLKPFERDISTEIYQAGSTPCNGVEGFNCYFPLQ | 0.3248 |
| 356-363 | KRISNCVA | 0.2218 |
| 525-534 | CGPKKSTNLV | 0.1904 |
| 341-354 | VFNATRFASVYAWN | 0.1552 |
| 673-682 | SYQTQTNSPR | -0.0008 |
