# Supplementary material for: Bioinformatic prediction of immunodominant regions in spike protein for early diagnosis of the severe acute respiratory syndrome coronavirus 2 (SARS-CoV-2)
Source: PeerJ. 2021 Apr 8;9:e11232. doi: 10.7717/peerj.11232 (PMC8038641; doi:10.7717/peerj.11232)
Supplement: Supplemental Information 4 [file peerj-09-11232-s004.docx]

| Position | Sequence | Allele | Antigenicity |
| --- | --- | --- | --- |
| 16-24 | VNLTTRTQL | H-2-Kb、H-2-Kd | 1.3468 |
| 24-32 | LPPAYTNSF | H-2-Dd | 0.3775 |
| 54-62 | LFLPFFSNV | H-2-Kb | -0.1776 |
| 60-68 | SNVTWFHAI | H-2-Kb | 0.4892 |
| 62-70 | VTWFHAIHV | H-2-Kb | 0.5426 |
| 84-92 | LPFNDGVYF | H-2-Ld | 0.5593 |
| 133-141 | FQFCNDPFL | H-2-Db | -0.2493 |
| 160-168 | YSSANNCTF | H-2-Db | -0.1036 |
| 168-176 | FEYVSQPFL | H-2-Kk | 0.6324 |
| 171-179 | VSQPFLMDL | H-2-Kb | 0.2718 |
| 202-210 | KIYSKHTPI | H-2-Kb | 0.7455 |
| 215-223 | DLPQGFSAL | H-2-Dd | 0.5622 |
| 223-231 | LEPLVDLPI | H-2-Kk | -0.0069 |
| 233-241 | INITRFQTL | H-2-Kb | 0.3934 |
| 262-270 | AAAYYVGYL | H-2-Kb | 0.4605 |
| 268-276 | GYLQPRTFL | H-2-Kd | 0.6082 |
| 312-320 | IYQTSNFRV | H-2-Kd | 0.3109 |
| 323-331 | TESIVRFPN | H-2-Kk | -0.6508 |
| 342-350 | FNATRFASV | H-2-Kb | 0.5609 |
| 350-358 | VYAWNRKRI | H-2-Kd | 0.5003 |
| 379-387 | CYGVSPTKL | H-2-Kd | 1.4263 |
| 447-455 | GNYNYLYRL | H-2-Kb | 0.117 |
| 464-472 | FERDISTEI | H-2-Kk | -0.7442 |
| 472-480 | IYQAGSTPC | H-2-Kd | -0.0319 |
| 484-492 | EGFNCYFPL | H-2-Kb、H-2-Ld | 0.5453 |
| 488-496 | CYFPLQSYG | H-2-Kd | 0.578 |
| 489-497 | YFPLQSYGF | H-2-Dd | 0.5107 |
| 503-511 | VGYQPYRVV | H-2-Kb | 1.4383 |
| 505-513 | YQPYRVVVL | H-2-Dd | 0.5964 |
| 510-518 | VVVLSFELL | H-2-Kb | 1.0909 |
| 525-533 | CGPKKSTNL | H-2-Dd | 0.1363 |
| 539-547 | VNFNFNGLT | H-2-Kb | 1.5069 |
| 612-620 | YQDVNCTEV | H-2-Db | 1.6172 |
| 643-651 | FQTRAGCLI | H-2-Kk | 1.7332 |
| 673-681 | SYQTQTNSP | H-2-Kd | 0.2919 |
