# Supplementary material for: Bioinformatic prediction of immunodominant regions in spike protein for early diagnosis of the severe acute respiratory syndrome coronavirus 2 (SARS-CoV-2)
Source: PeerJ. 2021 Apr 8;9:e11232. doi: 10.7717/peerj.11232 (PMC8038641; doi:10.7717/peerj.11232)
Supplement: Supplemental Information 5 [file peerj-09-11232-s005.docx]

| Position | Sequence | Allele | Antigenicity |
| --- | --- | --- | --- |
| 538-552 | CVNFNFNGLTGTGVL | H2-IAb | 1.3281 |
| 374-388 | FSTFKCYGVSPTKLN | H2-IAb | 1.0042 |
| 199-213 | GYFKIYSKHTPINLV | H2-Iab、H2-Ied | 0.9278 |
| 18-32 | LTTRTQLPPAYTNSF | H2-IAb | 0.79 |
| 60-74 | SNVTWFHAIHVSGTN | H2-IAb | 0.7044 |
| 263-277 | AAYYVGYLQPRTFLL | H2-Iab、H2-Ied | 0.6073 |
| 592-606 | FGGVSVITPGTNTSN | H2-IAb | 0.5825 |
| 238-252 | FQTLLALHRSYLTPG | H2-IEd | 0.5789 |
| 345-359 | TRFASVYAWNRKRIS | H2-Iab、H2-Ied | 0.4963 |
| 215-229 | DLPQGFSALEPLVDL | H2-IAb | 0.4812 |
| 140-154 | FLGVYYHKNNKSWME | H2-IEd | 0.4793 |
| 512-526 | VLSFELLHAPATVCG | H2-IAb | 0.4784 |
| 87-101 | NDGVYFASTEKSNII | H2-Iab、H2-Ied | 0.4277 |
| 52-66 | QDLFLPFFSNVTWFH | H2-IAb | 0.4159 |
| 233-247 | INITRFQTLLALHRS | H2-IAd | 0.4118 |
| 253-267 | DSSSGWTAGAAAYYV | H2-IAb | 0.3802 |
| 194-208 | FKNIDGYFKIYSKHT | H2-IEd | 0.3737 |
| 491-505 | PLQSYGFQPTNGVGY | H2-IAb | 0.3415 |
| 631-645 | PTWRVYSTGSNVFQT | H2-IAb | 0.2357 |
| 363-377 | ADYSVLYNSASFSTF | H2-IAb | 0.2252 |
| 350-364 | VYAWNRKRISNCVAD | H2-IEd | 0.1768 |
| 32-46 | FTRGVYYPDKVFRSS | H2-IEd | 0.1604 |
| 154-168 | ESEFRVYSSANNCTF | H2-IAb | 0.0628 |
| 450-464 | NYLYRLFRKSNLKPF | H2-IEd | 0.0415 |
| 445-459 | VGGNYNYLYRLFRKS | H2-IEd | -0.0193 |
| 37-51 | YYPDKVFRSSVLHST | H2-Iab、H2-Ied | -0.0237 |
| 469-483 | STEIYQAGSTPCNGV | H2-IAb | -0.0513 |
