# Supplementary material for: Bioinformatic prediction of immunodominant regions in spike protein for early diagnosis of the severe acute respiratory syndrome coronavirus 2 (SARS-CoV-2)
Source: PeerJ. 2021 Apr 8;9:e11232. doi: 10.7717/peerj.11232 (PMC8038641; doi:10.7717/peerj.11232)
Supplement: Supplemental Information 6 [file peerj-09-11232-s006.docx]

| Predicted epitope | IEDB ID | Sequence | MHC molecule | Assay description |
| --- | --- | --- | --- | --- |
| Spike _200-215_ | 1330367 | YFKIYSKHTPINLVRD | HLA class II | cellular MHC/mass spectrometry ligand presentation **Positive** |
| Spike_84-92_ | 1321049 | LPFNDGVYF | HLA-B*51:01  HLA-B*07:02  HLA-B*35:01 | biological activity activation **Positive** |
| Spike_202-210_ | 1319559 | KIYSKHTPI | HLA-B*08:01 | biological activity activation **Positive** |
| Spike_238-252_ | 1329417 | FQTLLALHRSYLTPG | HLA class II | cellular MHC/mass spectrometry ligand presentation **Positive** |
