# Supplementary figures and images for: Bioinformatic prediction of immunodominant regions in spike protein for early diagnosis of the severe acute respiratory syndrome coronavirus 2 (SARS-CoV-2)
Source: PeerJ. 2021 Apr 8;9:e11232. doi: 10.7717/peerj.11232 (PMC8038641; doi:10.7717/peerj.11232)

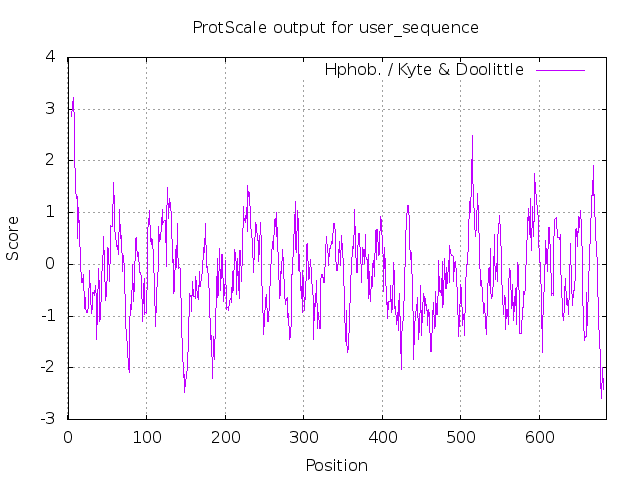

Supplement: Supplemental Information 7 — All prediction results including linear B-cell epitope, T-cell epitope, hydrophilicity and surface accessibility of SARS-CoV-2 spike protein S1 subunit. [file peerj-09-11232-s007.zip › Raw data/6. Hydrophilicity prediction results by protscale/Hydrophilicity prediction results by protscale.gif]
